# Retraction: Methionine-induced regulation of growth, secondary metabolites and oxidative defense system in sunflower (*Helianthus annuus* L.) plants subjected to water deficit stress

**DOI:** 10.1371/journal.pone.0274851

**Published:** 2022-09-30

**Authors:** The PLOS ONE Editors

The *PLOS ONE* Editors retract this article [1] because it was identified as one of a series of submissions for which we have concerns about authorship, competing interests, and peer review. We regret that the issues were not addressed prior to the article’s publication.

PA did not agree with the retraction. GM, NAA, MA, PK, and MAES either did not respond directly or could not be reached.
